# Complete genome sequence of new *Microbacterium* sp. strain ET2, isolated from roots of leafless orchid

**DOI:** 10.1128/mra.00899-23

**Published:** 2024-02-22

**Authors:** Elena A. Volynchikova, Maria G. Khrenova, Tatiana V. Panova, Vladimir A. Rodin, Maria I. Zvereva, Elena A. Tsavkelova

**Affiliations:** 1 Faculty of Biology, M.V. Lomonosov Moscow State University, Moscow, Russia; 2 Faculty of Chemistry, M.V. Lomonosov Moscow State University, Moscow, Russia; University of Strathclyde, Glasgow, United Kingdom

**Keywords:** orchid, plant-associated bacteria, nanopore sequencing

## Abstract

Whole-genome sequence of ET2 strain, isolated from the roots of leafless orchid, constitutes a single circular chromosome of 3,604,840 bp (69.44% G + C content). BLAST+-based average nucleotide identity (ANIb) and digital DNA-DNA hybridization values indicate that ET2 may be a novel *Microbacterium* species. Genes putatively involved in plant-microbial interactions were predicted.

## ANNOUNCEMENT

*Microbacterium* species inhabit diverse environments (1–5). For the isolation of *Chiloschista parishii*-associated bacteria from “Main Botanical Garden RAS” (Moscow; 55.83380, 37.59629), 50 µL of root suspension (0.1 g/10 mL water) originated from the two plants, were smeared onto R2A and incubated 5–7 days at 28°C. ET2 was selected and restreaked, and its 16S rRNA gene was sequenced with B63f-B1387r primers (6). It was deposited as Ac-2212 in VKPM (http://eng.genetika.ru/service-offer/vkpm/) and Ас−2998 in VKM (https://www.vkm.ru/).

For Illumina and Nanopore sequencing, DNA was extracted separately from colonies of ET2, cultured aerobically for 3 days (R2A, 30°C), using Monarch genomic DNA purification kit (NE BioLabs). Purified with Agencourt AMPure XP beads (Beckman Coulter), unsheared gDNA without size-selection was used for DNA library preparation according to Ligation sequencing gDNA (SQK-LSK109) protocol (ONT). Sequencing was conducted on “MinION” with R9.4.1 flow cell (ONT), resulting in 46,101 reads, containing 205,699,550 bases with N50/N90 values of 9,569/1,533 bp. Guppy basecaller (v.6.6.6) dna_r9.4.1_450bps_hac.cfg (7), with cutting Q at 7 for quality control was used.

The Illumina sequencing libraries were prepared by KAPA HyperPlus Library Preparation kit (Kapa Biosystems). Paired-end sequencing by Illumina NovaSeq 6000 produced 2 × 150 bp reads that were processed and filtered using Geneious Prime 2022.0.1. The genome assembled from Nanopore long-reads with Flye v.2.9.1 (8) into single circular chromosome was polished with medaka v.1.4.2 (https://github.com/nanoporetech/medaka) using default settings. Additional round of polishing was performed with Polypolish v.0.5.0 (9) using quality-filtered (*q* > 20) Illumina reads, yielding 668,741 paired reads as the final data set. Final assembly quality was determined by CheckM v.1.0.18 (10) and subjected to genome and functional annotation using NCBI PGAP v.6.5 (11) without rotation.

A single circular chromosome (69.44% G + C; 99.49% completeness; 0.63% contamination) of length 3,604,840 bp contained 3,325 protein-coding sequences (CDSs), 3 rRNA (1 5S, 1 16S, 1 23S), 48 tRNA, 3 non-coding RNA genes, 111 pseudogenes, and *dnaA* gene (origin of replication), all predicted by PGAP annotation.

ET2 may be a novel species according to ANIb and dDDH, calculated via JSpeciesWS service v3.9.0 (https://jspecies.ribohost.com/jspeciesws/) and the Type (Strain) Genome Server (12; https://tygs.dsmz.de/). The ANIb and dDDH values for the most closely related strains (13–15) were *Microbacterium kunmingense* (JAKNUS010000000)—86.2% and 32.6%, *Microbacterium saccharophilum* (BKAH00000000)—74.4% and 19.5%, *Microbacterium wangchenii* (CP038266)—74.9% and 20.0%, respectively (access date 02/2024).

Genes of isoprenoid (*idi*) and carotenoid (*ctrI*) pigment biosynthesis common for microbacteria (16) were predicted in the genome of untypically purple-colored strain ET2. Putative genes of plant growth promotion (17), encoding ACC deaminase and iron-siderophore complexes, phosphorus metabolism-related genes (18) *phoBU*, *pstABCS*, and that encoding inorganic phosphate transporter, as well as genes of indole-3-acetic acid (IAA) biosynthesis (19), encoding indole-3-acetaldehyde dehydrogenase and amidase (*amiE*), were predicted.

Publication of the genome of this novel species, which is expected to play an essential role in orchid-microbial interactions, contributes to our understanding of biodiversity.

## Data Availability

The whole-genome sequence of ET2 was deposited in GenBank (CP128170), together with the filtered SRA Illumina (SRR25019488) and Oxford Nanopore (SRR24955880) reads, attributed to the BioSample (SAMN35360712) and BioProject (PRJNA976223).
